# Study of Component Composition and Antimicrobial Activity of the Ophthalmic Emulsion Based on the Safflower Flowers (*Carthamus tinctorius L.*)

**DOI:** 10.1155/2022/3181270

**Published:** 2022-05-29

**Authors:** Zhanar Abuova, Aknur Turgumbayeva, Ardak Jumagaziyeva, Kairolla Rakhimov, Aigul Jussupkaliyeva

**Affiliations:** ^1^Department of Clinical Pharmacology, Asfendiyarov Kazakh National Medical University, Almaty, Kazakhstan; ^2^Department of Fundamental Medicine, Al-Farabi Kazakh National University, Almaty, Kazakhstan; ^3^Microbiology Laboratory, Scientific Center for Antiinfectious Drugs, Almaty, Kazakhstan

## Abstract

The use of medicinal plants has increased significantly in recent years. More than 80% of the world's population uses medicinal plants to treat themselves. Many antibacterial and anti-inflammatory synthetic drugs are available in medical practice. However, recent tendency of increasing capability of resistance of bacteria to usage of antibacterial drugs of different groups is taking place. Considering the wide range of pharmacological and antimicrobial activity of safflower flower extracts and available vitamins in their composition, it was decided to create a preparation based on the CO_2_ extract of safflower (*Carthamus tinctorius L.*) in the form of an ophthalmic emulsion. The aim of this research is to study the composition and antimicrobial activity of the extract and ophthalmic emulsion drops against test strains of microorganisms. The subject of this study is the ophthalmic emulsions from flowers of Kazakhstan species of “Akmai” safflower, collected in the flowering stage in southern Kazakhstan in August 2021. The component composition was determined using gas chromatography with the Agilent 7890A/5975C mass spectrometry technique. A study of the antimicrobial activity of the ophthalmic emulsion drop extracts was performed with two strains of Gram-positive bacteria, one strain of Gram-negative bacteria, and one culture of fungi. The following biologically active substances were determined from the GC-MS results: tridecane 94%, tricosane 93%, hexacosane 93%, dodecanoic acid 92%, pentacosane 91%, and linoleic acid 63.7%. The investigated emulsion-type eye drop shows bactericidal activity against *S. aureus* ATCC 6538-P, where the zone of growth suppression under the ophthalmic emulsion action corresponded to 9.0 ± 0.0 mm. The tested ophthalmic emulsion drops show the presumed biological activity against conditionally pathogenic bacteria. The results of chromatographic analysis and antimicrobial activity of the tested samples indicate the prospects for their further study for use as anti-infectious (anti-inflammatory) agents in medicine.

## 1. Introduction

According to the World Health Organization, within the next ten years, the share of drugs of plant origin in the medicinal supply of the population may be more than 80 percent [1]. There are more than six thousand plant species in the territory of the Republic of Kazakhstan, most of which can be used to produce domestic medicines [2]. Analysis of the development of phytopharmacology shows that the most promising direction in phytopreparation development is the scientifically validated use of the experience of folk and traditional medicines [3].

Among useful medicinal plants, the genus varieties of the *Carthamus tinctorius L.* occupy a special place. They belong to the *Compositae* family. They contain many biologically active substances: vitamins A and E, polyunsaturated fatty acids, linoleic acid (about 70%), monounsaturated oleic acid (10%) with small amounts of stearic acid, and other biologically active substances in large quantities. They have anti-inflammatory, antimicrobial, and antioxidant properties [4].

Safflower is best known as “kusum” (India and Pakistan), derived from Sanskrit “kusumbha,” and as “hongkhua” (red flower) in China. Its use as a less-expensive substitute for saffron is indicated by the names false saffron, bastard saffron, thistle saffron, and saffron dye [5]. The medicinal properties of the herb *Carthamus tinctorius L.* have been known since ancient times and have been used for centuries in traditional and folk medicine around the world. Safflower eye drops reduce myopia, especially in children. Trachoma has been successfully treated with safflower combined with other herbs. Invigoration of the blood circulation by safflower has also reduced senile cataracts [6, 7]. The flower extract was found to increase antibacterial activity, decrease depression symptoms, relieve inflammation, and retard the progression of skin tumors [8].

More than 104 compounds have been isolated and identified from this plant, and quinochalcones and flavonoids are considered characteristics and active components of the safflower [9]. Targeted identification of potentially biologically active molecules from herbal medicines is often challenging. It might be because of insufficient chromatographic separation, ubiquitous matrix interference, and common isomerism. An enhanced targeted identification strategy is presented and validated by the selective identification of flavonoid O-glycosides (FOGs) from *Carthamus tinctorius* [10]. More than 200 different compounds isolated from *C. tinctorius L.*, including fatty acids, steroids, flavonoids, coumarins, and polysaccharides, have antimicrobial properties [11, 12]. The results are in accordance with Hiramatsu et al. who observed antioxidant and neuroprotective activities of *Carthamus tinctorius*, which suggest that the petal extract of *Carthamus tinctorius* has free radical scavenging activity and neuroprotective effects. Carthamin is also one of the major active components [8].

## 2. Materials and Methods

### 2.1. Plant Material

Flowers of the Kazakhstan safflower species were collected in the South Kazakhstan region in the flowering phase in August 2021. The plant was identified by Konyrbekov M., the taxonomist of the station. The voucher specimen was transferred for storage to the herbarium of the Krasnovodopadskaya Experimental Breeding Station, Ministry of Agriculture, Republic of Kazakhstan.

### 2.2. Obtaining Carbon Dioxide Extract

The extract was obtained from the *Carthamus tinctorius*, a 1000 g dried plant, under subcritical conditions of CO_2_ extraction for 13 hours at 45–52 atm and 19–22 C to obtain a dark brown-colored extract. The purpose of *Carthamus tinctorius L.* extraction is to obtain large amounts of an extract rich in the desired active compounds in a time-sensitive and cost-effective manner. The productivity and profitability of a subcritical CO_2_ extraction process largely depended on the selection of process parameters. Carbon dioxide (CO_2_) is the most desirable solvent for the subcritical extraction of natural products. Its near-ambient critical temperature makes it suitable for the extraction of thermolabile components without degradation.

### 2.3. Component Composition Determination

Gas chromatography with Agilent 7890A/5975C mass spectrometric detection was used to conduct the extract composition analysis. Chromatography conditions were as follows: sample 1.0 *μ*l, sample input temperature 260°C, and no flow splitting. Separation was performed using a DB-WAXetr chromatographic capillary column with a 30 m length, an inner diameter of 0.25 mm, and a film thickness of 0.25 *μ*m at a constant carrier gas rate (helium) of 1 ml/min. [13]. The chromatography temperature is programmed from 40°C (10 min time of exposure) at a heating rate of 5°C/min to 270°C (10 min exposure). Detection is performed in scan mode m/z 34–750. Agilent MSD ChemStation software (version 1701EA) was used to control the gas chromatography system, record, and process the results and data [14]. The chromatogram obtained was analysed, and each peak was checked by determining the percent area on the chromatogram, the retention time, the spectrum, and the base peak and then referring to the characteristic mass spectra of compounds listed.

### 2.4. Component Identification

The Wiley 7th edition and NIST'02 library used to identify the mass spectra obtained. The percentage of components was calculated automatically based on the peak areas of the total ion chromatogram. The component identification was The components were identified by mass spectra and retention times.

### 2.5. Study of Antimicrobial Activity

Determination of antimicrobial activity was carried out by double serial dilutions in a liquid nutrient medium according to the international standard CLSI M100-S25, 2015, “*Performance Standards for Antimicrobial Susceptibility Testing*,” M100-S25, 2015, Vol. 35, No. 3, and the methodological guidelines “Methodological Guidelines for Determining the Sensitivity of Microorganisms to Antibacterial Agents,” MUK 4.12.1890–04, Moscow, 2004. Antifungal activity determination was carried out in accordance with the international standard CLSI M27-A2: “*Reference Method for Broth Dilution Antifungal Susceptibility Testing of Yeast*,” Vol. 22, No. 15 [15–19].

#### 2.5.1. Micromethod of Serial Dilutions

A 48-well plate was used to determine antimicrobial activity. 1 to 11 wells were filled with 0.5 mL of the MHB broth (for testing bacteria) or Saburo broth (for testing fungi). The working solution (in this case, it is the initial extract), was added in pure form (0.5 ml) to the 1st test tube. Next, serial dilutions were performed by adding the mixture (MHB (0.5 ml) + test drug (0.5 ml)) from the 2nd test tube at an amount of 0.5 ml into the 3rd test tube already containing 0.5 ml of the broth. 0.5 ml of the test sample was thoroughly mixed and transferred to the broth from the 3rd vial to the 4th tube containing 0.5 ml of broth initially. This procedure was repeated until the required number of dilutions was obtained. From the last tube, 0.5 ml of the mixture was removed. Thus, the following dilutions were obtained: 1 : 1, 1 : 2, 1 : 4, 1 : 8, 1 : 16, 1 : 32, 1 : 64, 1 : 128, 1 : 256, 1 : 512, and 1 : 1024, corresponding to tubes 1 through 11, well 12 being the culture growth control.

After a series of dilutions, 0.05 mL of the test strain was added to all wells at the appropriate concentration. The procedure was repeated for all test samples and test strains.

All samples were incubated for 18–24 hours at 37 ± 1°C. At the end of the incubation time, seeding was performed on Petri dishes to determine live cells. After seeding, the dishes were placed in a thermostat for 18–24 hours at 37 ± 1°C.

The results were counted by the presence of visible growth of microorganisms on the surface of dense nutrient media. The minimum bactericidal dilution was the lowest in the well that suppressed the growth of microorganisms.

#### 2.5.2. Disco-Diffuse Method

A DEN-1 densitometer designed to measure optical density (turbidity) was used to prepare microbial suspensions with the required concentration. Sodium chloride physiological solution (0.9% NaCl) was used to prepare the suspension. 5 ml of physiological solution was added to a test tube and placed in a densitometer, and optical density was measured.

First, a suspension was prepared with a concentration of 1.5 × 108 CFU/ml. This concentration corresponds to a turbidity of 0.5 McFarland units for bacteria and 2.5 for fungi. From these suspensions, 10-fold dilutions were made by transferring 1.0 mL of suspension to 9.0 mL of sterile saline. Thus, dilutions of 1.5 × 106 CFU/ml for bacteria and 7.5 × 106 CFU/ml for fungi were obtained. The CO_2_ extract and ophthalmic emulsion were also tested by the alveolar method. For this purpose, wells with 6 mm diameter were made in agar seeded with test microorganism strains. Extract solutions were added to the wells at a volume of 200 *μ*l. The results were recorded by measuring the diameter of culture growth suppression around the wells using a ruler as calipers. The results were used to calculate the mean values and standard deviation (SD) using MS-Office Excel software. The results were interpreted according to the CLSI standard.

## 3. Results and Discussion

### 3.1. Results of Extraction

The CO_2_ extract was obtained by subcritical carbon dioxide extraction (installation of carbon dioxide flow—through extraction—5L). The resulting extract yield was 10 g. CO_2_ extract from the *Carthamus tinctorius L.* was developed and obtained at ZhanaPharm LLP in Kazakhstan.

The subcritical CO_2_ extraction has a high extraction rate, short extraction time, and clarity product. Its usage to obtain bioactive compounds of *Carthamus tinctorius L.* has been shown to be a promising alternative to conventional extraction methods since it allowed the extraction of compounds with scientific and industrial interest.

### 3.2. Results of Chromatographic Analysis

**Figure 1:**
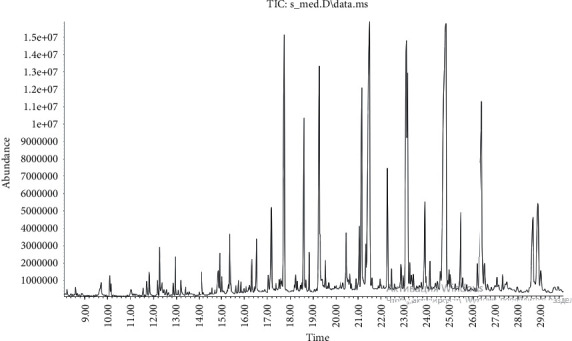
Chromatogram of the CO_2_ extract of safflower.

The compounds of the obtained extract are listed in Table 1, Table S2 (Supplementary Materials), and Table 2. The obtained extract was found to be rich in linoleic acid, octane, 2-nonen-1-ol, hexadecanal, dodecanal, dec-2-en-1-ol, nonanoic acid, tetradecanoic acid, 2 pentadecanone, 6,10,14-trimethyl, 1,2-benzenedicarboxylic acid, isobutyl-beta-phenylpropionate, octadecanoic acid, heneicosanoic acid, 2(3H)-furanone, 4,4-dipropylheptane, hexacosane, tocopherol, 1-eicosanol, tricosane, and tetracosane. The mass spectrum of the CO_2_ extract showed the peak at which 85 compounds were found (Figure 1).

### 3.3. Results of Developed Ophthalmic Emulsion

Ten models were developed (Table 3). Nine models did not meet the requirements: the first model turned out to be a heterogeneous mass with yellow lumps; the second one was a very thick heterogeneous yellow mass, and oil stains were observed on the walls of the containers; the third one was a heterogeneous yellow mass, etc. The most optimal composition turned out to be the tenth model.

The optimal model was selected based on the most appropriate parameters: white homogenous liquid; quickly gets absorbed; not spreading when applied to glass (shows the presence of oil extract); and flaky, but easily reconstituted when shaken. Ten models of the emulsion-type eye drop based on the oil extract of safflower (*Carthamus tinctorius L.*) with vasodilator effect with various excipients were developed: as a conservant, citric acid was used, as a prolongator, methylcellulose and sodium carboxymethyl cellulose were used, as an emulsifier, Tween-80 and gelatose were used, and water for injection was used as a solvent. Parameters of the emulsion-type eye drops were examined by organoleptic and microscopic methods.

The composition of the tenth model is as follows: active substance was the CO_2_ extract (0.85 ml) and excipients were citric acid (0.04 g), methylcellulose (0.04 g), Tween-80 (0.15 g), and water for injection (up to 10 ml). Manufacturing technology was as follows: (1) the safflower extract and Tween-80 were intensively mixed (condition: temperature has to be 60–70°C); (2) methylcellulose and water for injection were slowly mixed (condition: temperature has to be 50°C; requirement: no bubbles); (3) mixtures from steps 1 and 2 were homogenised (condition: temperature has to be 50°C).

### 3.4. Results of Antimicrobial Activity

The results of the study of antibacterial and fungicidal activity of the extract and the emulsion-type eye drop against strains of pathogenic microorganisms *S. aureus* ATCC 6538-P, *E. coli* ATCC 8739, *P. aeruginosa* ATCC 9027, and *C. albicans* ATCC 10231 are presented in Table 1.

From the data presented in Table 4 (serial dilution method), we can see that the extract exhibits the assumed biological activity against the strains tested. Thus, concerning *S. aureus* ATCC 6538-P, the extract shows the bacteriostatic effect in dilution of 27.03 mg/ml and the extract shows the bacteriostatic effect concerning *Pseudomonas bacillus* in dilution of 54.05 mg/ml. The fungistatic effect of the extract concerning the *C. albicans* test strain ATCC 10231 was recorded in dilution of 13.5 mg/ml. The ophthalmic emulsion showed no bactericidal effect against the *E. coli* test strain ATCC 8739.

From the data presented in Table 5 (diffusion method), we can see that the investigated emulsion-type eye drop shows bactericidal activity against *S. aureus* ATCC 6538-P, where the zone of growth suppression under the emulsion-type eye drop action corresponded to 9.0 ± 0.0 mm. The fungicidal effect of this extract against the test strain *C. albicans* ATCC 10231 was not recorded. The extract showed no antimicrobial action against *S. aureus* ATCC 6538-P and *C. albicans* ATCC 10231.

The conducted experiments showed the possibility of practical use of *Carthamus tinctorius L.,* which will expand the range of medicinal plant materials. Flavonoid and oil extracts from safflower had good antibacterial activity against *E. coli* and *S. aureus*. Results support the observation that Gram-negative bacteria are more resistant probably because of their thick murein layer, which prevents the penetration of inhibitors due to the fact that the cell membrane of Gram-negative bacteria consists of many condensed lipid fat layers compared to the membrane of Gram-positive cells [20]. In the works of the authors Sabah and Salieh, different concentrations of the flavonoid extracts of safflower flowers (25 *μ*g/ml, 75 *μ*g/ml, 250 *μ*g/ml, 500 *μ*g/ml, 1000 *μ*g/ml, and 100000 *μ*g/ml) tested in the experiment were much effective compared to the same concentrations of the oil extract and the flavones tested produced varied inhibition zones. And they suggested that the flavones with more number of –OH groups inhibited both Gram-positive bacteria and Gram-negative bacteria, thereby suggesting a broad-spectrum antibacterial activity [21]. Karimkhani, Shaddel, Khodaparast, Vazirian, and Piri Gheshlaghi studied antioxidant and antimicrobial activities in safflower methanolic extracts of different cultivars (IL111, Padide, Isfahan-28, and Mahali). The Isfahan-28 cultivar showed the best antimicrobial activity; the minimum inhibitory concentration against *Staphylococcus aureus* and *Salmonella enterica serovar Typhi* was 30 and 60 mg/ml, respectively [22]. On the other hand, safflower is susceptible to several fungal diseases such as Alternaria, wilt, and rust. Thus, plant extract use with antifungal power is a promising alternative against these diseases [23]. Previous studies have shown that the safflower water extract has antibacterial properties against various bacteria such as *Bacillus subtilis*, *Bacillus cereus*, and *Bacillus mycoides* [24]. Another study showed that safflower methanolic and water extracts have a high potency in inhibiting the growth of *S. aureus*, *E. coli*, *P. aeruginosa*, and *K. pneumonia* bacteria and their inhibitory power varies depending on the type of bacteria [25]. The antibacterial activity of plant extracts is more related to the presence of polyphenolic compounds. These compounds inhibit bacterial growth through various mechanisms such as cell wall destruction, cell membrane destruction, and intracellular bacterial matrix destruction. Bacterial type, cell wall structure, and quantitative and qualitative content of extract compounds are among the determinants of the extract's antibacterial potency [26–29].

The study of Aditya et al. showed the antibacterial properties of dehydroabietylamine isolated from *Carthamus tinctorius L.* followed by the in silico elucidation of its probable mode of action. The minimum inhibition concentration (MIC) and minimum bactericidal concentration (MBC) of dehydroabietylamine were assessed against *Staphylococcus aureus* and *Pseudomonas aeruginosa*, using the microbroth dilution method. The dehydroabietylamine established the significant MIC for *S. aureus* (12.5 *μ*g/ml) and *P. aeruginosa* (6.25 *μ*g/ml), respectively [30]. However, Salem et al. reasoned the antimicrobial activity of precarthamin and carthamin, and the quantitative variation of these molecules could be due to a colour change in *C. tinctorius* flowers. Therefore, during flowering, the antimicrobial activity of these two natural dyes increased where the inhibition zone (iz) of carthamin reached up to 25.89 mm mainly against *E. coli* at the increased frequency of resistance to commonly used antibiotics leads to the search for new effective natural drugs in food and pharmaceutical industries [31].

As it was seen in the previous literature, the compounds including hexadecanoic acid, octadecenoic acid, stigmasterol, and *γ*-sitosterol may inhibit the pathogenic bacteria. This is supported by several researchers. 9-Octadecanoic acid and hexadecanoic acid identified from neem oil had effectively inhibited the pathogenic bacteria, including *Staphylococcus aureus* ATCC No. 25923, *Escherichia coli* ATCC No. 44102, and *Salmonella sp.* ATCC No. 50041 by in vitro antibacterial screening [32]. Antibacterial activity of compounds was related to the amount and type of unsaturated bonds, so compounds that contained unsaturated double bonds, such as alcohols, aldehydes, ketones, acids, and terpenes, might play a significant role in antibacterial effects [33]. Cui et al. noted in their research that at a concentration of 20 mg/mL, tricosane was active against all microorganisms, with inhibition zones ranging from 8.03 to 15.97 mm. They proved that tricosane showed a concentration-related increase in inhibition zones against *P. fluorescens* SHL7, *E. coli*, *B. subtilis*, and *S. cerevisiae* Ja-64 and Y-8. *Bacillus subtilis* was the most sensitive strain with the largest inhibition zones (7.20–15.97 mm) [34].

**Figure 2:**
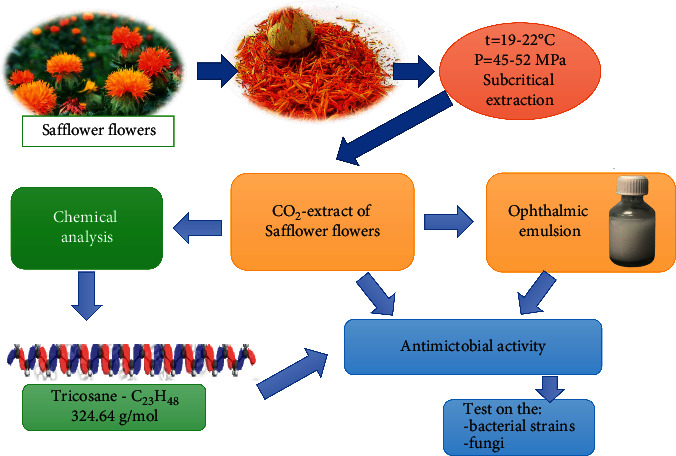
Safflower flowers, ophthalmic emulsion, and their possible antimicrobial effects.

However, the antimicrobial activity of the safflower extract and ophthalmic emulsion may be connected to tricosane content, which is higher in the chromatographic analysis (Figure 2).

Thus, it was first established that ophthalmic emulsion based on the safflower flowers *(Carthamus tinctorius L.),* growing in the territory of Kazakhstan has antimicrobial activity.

## 4. Conclusions

The study results of the component composition of the CO_2_ extract of *Carthamus tinctorius L.* are presented above. The main components in the raw material are terpenoids, sterols, fatty acids, and tocopherols. The chromatographic analysis results will be used for standardization of the preparation.

Ten models using different bases were developed, and the optimal model no. 10 was selected. The full composition is outlined in the results of the study. Research in the development of the composition and technology of eye drops based on the safflower extract is planned to continue in the future. The tested extract and ophthalmic emulsion exhibit the alleged biological activity against conditionally pathogenic bacteria and the genus *Candida* fungi. The results of studied samples' bacteriostatic and fungistatic activity indicate the prospects for further study for use as anti-infectious agents in medicine. *Staphylococcus aureus* is an increasingly common pathogen in ophthalmology. The ophthalmic emulsion development based on the plant origin is an important task.

## Figures and Tables

**Table 1:** Results of chromatographic analysis of the CO_2_ extract of safflower.

#	RT	RI calc	RI lit	Compound	Variability	Area %
1	11.99	948	958 ± 6	Palmitic acid (16 : 0)	927	0.5
2	14.672	990	1012 ± 4	Hexadecenoic acid (16 : 1)	903	1.1
3	26.214	1135	1142 ± 3	Stearic acid (18 : 0)	956	15.3
4	36.677	1254	1263 ± 3	Octadecenoic acid (18 : 1)	896	10.1
5	39.461	1288	1317 ± 3	Linoleic acid (18 : 2)	890	63.7
6	41.435	1312	1317 ± 9	Linolenic acid (18 : 3)	879	1.2
7	47.591	1385	1404 ± 7	Octadecatrienoic acid (18 : 3)	945	0.2
8	48.439	1395	1402 ± 3	Arachidic acid (20 : 0)	939	3.2
Total						95.3

**Table 2:** Results of chromatographic analysis.

#	RT	RI calc	RI lit	Compound	Variability	Area %
1	117.601	3116	3138 ± 8	Vitamin E	929	3.3

**Table 3:** Figures of model mixtures of emulsion-type eye drop based on the СО_2_ extract of safflower (*Carthamus tinctorius L.)*.

Model no. 1	Model no. 2	Model no. 3	Model no. 4	Model no. 5
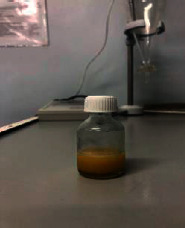	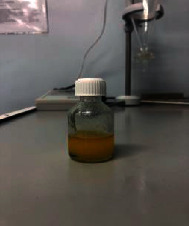	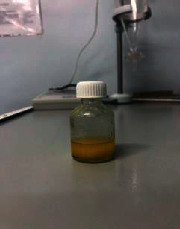	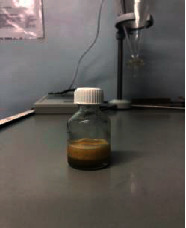	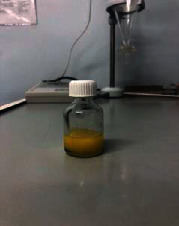
Model no. 6	Model no. 7	Model no. 8	Model no. 9	Model no. 10
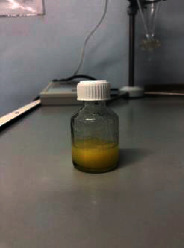	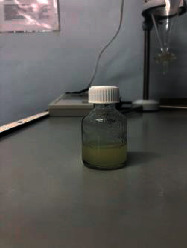	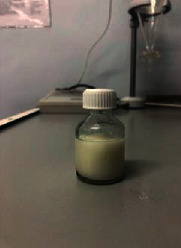	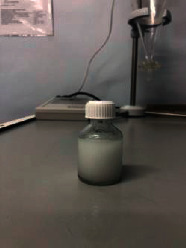	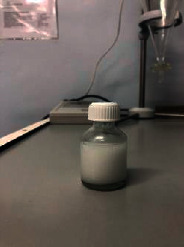

**Table 4:** Results of antimicrobial activity of the extract and eye drops obtained by serial dilution.

Test sample	Minimum bactericidal concentration (extract dilution)			
	*S. aureus* ATCC 6538-Р	*E. coli* ATCC 8739	*P. aeruginosa* ATCC 9027	*C. albicans* ATCC 10231
Extract no. 1	27.03^*∗*^	—	54.05^*∗*^	13.5^*∗*^
Ophthalmic emulsion	54.05	27.03^*∗*^	27.03^*∗*^	—

*Note.* The symbol “—” indicates the extract does not have antimicrobial activity and “^*∗*^” indicates the extract has bacteriostatic effect.

**Table 5:** Results of antimicrobial activity of extracts obtained by the diffusion method.

Test sample	Growth suppression zone, mm	
	*S. aureus* ATCC 6538-Р	*C. albicans* ATCC 10231
CO_2_ extract	6.0 ± 0.0	6.0 ± 0.0
Ophthalmic emulsion	9.0 ± 0.0	6.0 ± 0.0

## Data Availability

The data used to support the findings of this study are included within the article.

## Supplementary Materials

Supplementary Materials Results of chromatographic analysis of organic compounds in the plant extract of safflower are shown in Table S2.
