# Supplementary material for: Study of Component Composition and Antimicrobial Activity of the Ophthalmic Emulsion Based on the Safflower Flowers (Carthamus tinctorius L.)
Source: Int J Microbiol. 2022 May 29;2022:3181270. doi: 10.1155/2022/3181270 (PMC9168215; doi:10.1155/2022/3181270)
Supplement: Supplementary Materials — Results of chromatographic analysis of organic compounds in the plant extract of safflower are shown in Table S2. [file 3181270.f1.docx]

Supplementary material

Table 2. Results of chromatographic analysis of organic compounds in the plant extract of *Safflower*

| № | Retention time, min | Compound | Probability of identification, % | Percentage, % |
| --- | --- | --- | --- | --- |
| 1 | 8.24 | 2-Octenal, (E)- | 87 | 0.09 |
| 2 | 8.40 | 2-Nonanone | 83 | 0.04 |
| 3 | 8.61 | Nonanal | 82 | 0.13 |
| 4 | 8.67 | Dodecane | 86 | 0.04 |
| 5 | 9.72 | Octanoic acid, ethyl ester | 88 | 0.45 |
| 6 | 10.10 | Decanal | 92 | 0.27 |
| 7 | 10.15 | Tridecane | 94 | 0.15 |
| 8 | 11.03 | Nonanoic acid | 83 | 0.20 |
| 9 | 11.55 | Tetradecane | 86 | 0.11 |
| 10 | 11.71 | 2-Sec-Butylcyclohexanone | 72 | 0.25 |
| 11 | 11.82 | 2,4-Decadienal, (E,E)- | 86 | 0.44 |
| 12 | 12.18 | Pentadecane, 2,6,10,14-tetramethyl- | 86 | 0.21 |
| 13 | 12.27 | 2,4-Decadienal, (E,E)- | 93 | 0.71 |
| 14 | 12.37 | n-Decanoic acid | 90 | 0.36 |
| 15 | 12.46 | Decanoic acid, ethyl ester | 78 | 0.11 |
| 16 | 12.56 | β-Maaliene | 82 | 0.11 |
| 17 | 12.87 | Pentadecane | 84 | 0.20 |
| 18 | 12.96 | 1-Pentadecene | 92 | 0.45 |
| 19 | 13.08 | Cedr-8(15)-ene | 83 | 0.11 |
| 20 | 13.20 | 4-Nonanone, 7-ethyl- | 72 | 0.31 |
| 21 | 13.40 | 4,4,6-Trimethyl-cyclohex-2-en-1-ol | 71 | 0.13 |
| 22 | 14.00 | Eudesma-4(14),11-diene | 76 | 0.08 |
| 23 | 14.15 | Tridecanal | 92 | 0.31 |
| 24 | 14.60 | 3-Buten-2-one, 4-(2,2,6-trimethyl-7-oxabicyclo[4.1.0]hept-1-yl)- | 80 | 0.14 |
| 25 | 14.87 | Dodecanoic acid | 92 | 0.67 |
| 26 | 14.94 | Dodecanoic acid, ethyl ester | 88 | 0.59 |
| 27 | 15.02 | Nonanoic acid, 9-oxo-, ethyl ester | 90 | 0.32 |
| 28 | 15.37 | Tetradecanal | 93 | 1.29 |
| 29 | 15.64 | 9,12,15-Octadecatrien-1-ol, (Z,Z,Z)- | 78 | 0.22 |
| 30 | 15.74 | Caryophyllene oxide | 89 | 0.29 |
| 31 | 15.86 | Phytol | 73 | 0.30 |
| 32 | 16.00 | Tridecanoic acid | 82 | 0.17 |
| 33 | 16.09 | Ethyl tridecanoate | 86 | 0.16 |
| 34 | 16.24 | Fumaric acid, ethyl 2-methylallyl ester | 75 | 0.37 |
| 35 | 16.33 | 2(4H)-Benzofuranone, 5,6,7,7a-tetrahydro-4,4,7a-trimethyl-, (R)- | 77 | 0.60 |
| 36 | 16.45 | Octadecane | 85 | 0.14 |
| 37 | 16.53 | Pentadecanal- | 92 | 1.04 |
| 38 | 17.05 | 3,7,11,15-Tetramethyl-2-hexadecen-1-ol | 85 | 0.32 |
| 39 | 17.18 | Tetradecanoic acid, ethyl ester | 80 | 1.98 |
| 40 | 17.53 | Eicosane | 85 | 0.38 |
| 41 | 17.59 | Phytol, acetate | 77 | 0.46 |
| 42 | 17.74 | 2-Pentadecanone, 6,10,14-trimethyl- | 92 | 5.59 |
| 43 | 18.24 | Pentadecanoic acid, ethyl ester | 91 | 0.52 |
| 44 | 18.31 | Phytol | 73 | 0.31 |
| 45 | 18.45 | Ethyl 15-methyl-hexadecanoate | 65 | 0.25 |
| 46 | 18.60 | Methyl 3,5-tetradecadiynoate | 66 | 3.18 |
| 47 | 19.26 | Hexadecanoic acid, ethyl ester | 87 | 5.11 |
| 48 | 19.54 | Heneicosane | 92 | 0.70 |
| 49 | 19.69 | Octadecanal | 84 | 0.33 |
| 50 | 20.20 | Heptadecanoic acid, ethyl ester | 80 | 0.61 |
| 51 | 20.34 | Phthalic acid, butyl dodecyl ester | 85 | 0.40 |
| 52 | 20.49 | Docosane | 88 | 1.34 |
| 53 | 20.58 | Hexadecanoic acid, octadecyl ester | 65 | 0.27 |
| 54 | 20.65 | Octadecanal | 80 | 0.41 |
| 55 | 20.71 | Phytol, acetate | 82 | 0.20 |
| 56 | 20.92 | 1-Hexadecyn-3-ol, 3,7,11,15-tetramethyl- | 74 | 0.30 |
| 57 | 21.06 | Ethyl Oleate | 91 | 1.27 |
| 58 | 21.16 | 9,12-Octadecadienoic acid, ethyl ester | 90 | 4.61 |
| 59 | 21.35 | Ethyl 9,12,15-octadecatrienoate | 88 | 0.76 |
| 60 | 21.50 | Tricosane | 93 | 10.81 |
| 61 | 21.61 | Hexadecanal | 81 | 0.44 |
| 62 | 21.96 | 2H-Pyran-2-one, tetrahydro-6-nonyl- | 63 | 0.68 |
| 63 | 22.28 | Tetracosane | 92 | 2.06 |
| 64 | 22.46 | Oxirane, heptadecyl- | 85 | 0.55 |
| 65 | 22.87 | Methyl 19-methyl-eicosanoate | 87 | 0.94 |
| 66 | 22.97 | 2-Cyclohexen-1-one, 4-hydroxy-3,5,5-trimethyl-4-(3-oxo-1-butenyl)- | 64 | 0.32 |
| 67 | 23.16 | Pentacosane | 91 | 4.21 |
| 68 | 23.25 | 4,8,12,16-Tetramethylheptadecan-4-olide | 86 | 0.61 |
| 69 | 23.33 | 1,22-Docosanediol | 78 | 0.41 |
| 70 | 23.41 | Thiophene, 3-methyl-2-pentadecyl- | 70 | 0.49 |
| 71 | 23.91 | Hexacosane | 93 | 2.15 |
| 72 | 24.37 | 9,12-Octadecadienoic acid, 2-phenyl-1,3-dioxan-5-yl ester, cis- | 69 | 0.48 |
| 73 | 24.46 | Docosanoic acid, ethyl ester | 71 | 0.37 |
| 74 | 24.82 | Triacontane | 92 | 16.28 |
| 75 | 24.91 | 13-Methylheptacosane | 79 | 0.19 |
| 76 | 25.02 | Phthalic acid, di(2-propylpentyl) ester | 88 | 0.53 |
| 77 | 25.23 | Hexacosane, 9-octyl- | 72 | 0.27 |
| 78 | 25.46 | Octacosane | 92 | 1.43 |
| 79 | 25.55 | 2-Pentacosanone | 76 | 0.46 |
| 80 | 25.73 | (Z)-14-Tricosenyl formate | 75 | 0.21 |
| 81 | 26.42 | Nonacosane | 93 | 8.15 |
| 82 | 26.55 | 3,6-Nonadecadione | 65 | 0.74 |
| 83 | 27.34 | Triacontane | 89 | 0.39 |
| 84 | 28.66 | Hentriacontane | 88 | 2.92 |
| 85 | 28.88 | Tricosane-2,4-dione | 61 | 3.10 |
